# Complement component 3 protects human bronchial epithelial cells from cigarette smoke-induced oxidative stress and prevents incessant apoptosis

**DOI:** 10.3389/fimmu.2022.1035930

**Published:** 2022-12-20

**Authors:** Yuqiang Pei, Jing Zhang, Jingge Qu, Yafei Rao, Danyang Li, Xiaoyan Gai, Yahong Chen, Ying Liang, Yongchang Sun

**Affiliations:** Department of Respiratory and Critical Care Medicine, Peking University Third Hospital, Research Center for Chronic Airway Diseases, Peking University Health Science Center, Beijing, China

**Keywords:** complement C3, chronic obstructive pulmonary disease, cigarette smoke, apoptosis, oxidative stress

## Abstract

The complement component 3 (C3) is a pivotal element of the complement system and plays an important role in innate immunity. A previous study showed that intracellular C3 was upregulated in airway epithelial cells (AECs) from individuals with end-stage chronic obstructive pulmonary disease (COPD). Accumulating evidence has shown that cigarette smoke extract (CSE) induces oxidative stress and apoptosis in AECs. Therefore, we investigated whether C3 modulated cigarette smoke-induced oxidative stress and apoptosis in AECs and participated in the pathogenesis of COPD. We found increased C3 expression, together with increased oxidative stress and apoptosis, in a cigarette smoke-induced mouse model of COPD and in AECs from patients with COPD. Different concentrations of CSEinduced C3 expression in 16HBE cells *in vitro*. Interestingly, C3 knockdown (KD) exacerbated oxidative stress and apoptosis in 16HBE cells exposed to CSE. Furthermore, C3 exerted its pro-survival effects through JNK inhibition, while exogenous C3 partially rescued CSE-induced cell death and oxidative stress in C3 KD cells. These data indicate that locally produced C3 is an important pro-survival molecule in AECs under cigarette smoke exposure, revealing a potentially novel mechanism in the pathogenesis of COPD.

## 1 Introduction

Chronic obstructive pulmonary disease (COPD) is a chronic and progressive inflammatory pulmonary disease characterised by persistent airflow limitation and respiratory symptoms. COPD is a major cause of morbidity and mortality, being the third leading cause of death globally in 2019 (1, 2). The major risk factor for COPD is exposure to cigarette smoke (3), a highly complex mixture of gases and particles with oxidative and carcinogenic properties that induces chronic inflammation, protease/antiprotease imbalance, and nuclear factor kappa B (NF-κB)-activated oxidative stress in the lung tissue (4). Oxidative stress is an important mechanism in COPD pathogenesis (5). Oxidants can be produced both by cigarette smoke and by activated inflammatory cells such as macrophages and neutrophils (6, 7). The transcription factor nuclear factor erythroid 2-related factor (Nrf2), a regulator of antioxidant genes and endogenous antioxidants, is reduced in patients with COPD (8). Studies have shown that oxidative stress-related markers, such as reactive oxygen species (ROS) and malondialdehyde (MDA) levels, are significantly elevated in smokers or patients with COPD, while antioxidants such as glutathione, superoxide dismutase (SOD) and catalase (CAT) are significantly reduced (9).

Cigarette smoke-induced COPD is also associated with dysregulated cell death processes (10). Previous studies found that apoptosis of both airway epithelial cells (AECs) and endothelial cells was significantly increased in COPD (11, 12). AECs from patients with COPD exhibit a constitutional pro-inflammatory phenotype and increased sensitivity to apoptosis. However, the molecular pathways involved in regulating apoptosis in COPD need to be further investigated.

The complement system plays a crucial role in the immune system in both innate and adaptive immune responses (13). As the most abundant complement protein in the blood, complement component 3 (C3) is a key player in the complement system and a vital factor in the innate immune system. It plays a significant role in the detection and clearance of potential pathogens by binding to other complement proteins (14). Circulating C3 is produced primarily in the liver; however, some immune cells and non-immune cells, such as lymphocytes, neutrophils and mesenchymal cells, can also synthesize C3 (15, 16). More recent studies have found that human AECs have biosynthetically-derived intracellular stores of C3 and that internalised C3 protects AECs from stress-induced death (17). Other studies have indicated that intracellular C3 protects rodent and human pancreatic β-cells from cytokine-induced apoptosis (18). C3 is highly expressed in isolated human islets, and C3-knockout β-cells exhibit increased cell death after challenge with diabetogenic stresses (19). More importantly, intracellular C3 was found to be present at high levels in AECs from individuals with COPD (17). These findings suggest that intracellular C3 may have functions other than defence against infection.

In the current study, we examined the expression of C3 in COPD and explored its role in COPD pathogenesis. We found that AECs from mouse and human COPD patients expressed a higher amount of C3 and that *in vitro* CSE stimulation upregulated C3 expression in AECs. Interestingly, C3 knockdown in AECs increased oxidative stress and apoptosis upon CSE exposure, whereas treatment with exogenous C3 attenuated CSE-induced death of AECs after C3 knockdown. These data revealed a new role for C3 as a pro-survival protein in human AECs exposed to CSE-induced stress in COPD.

## 2 Materials and methods

### 2.1 Human samples

The ethics review committee of Peking University Third Hospital approved the human study (batch number: S2018193), and the duration of the approved permit was 4 years, starting from August 2018. Informed consent was obtained from all participants. The human research complied with the Declaration of Helsinki. Human lung samples were obtained from patients with solitary tumours resected surgically, and lung tissues at a maximum distance from the tumour were collected by a pathologist. Thirty subjects were recruited and divided into a healthy non-smoking (HNS) group, a smoker group, and a COPD group. HNS controls were defined as never-smokers having a post-bronchodilator FEV_1_/FVC ≥ 0.7. Smokers were defined as those with a post-bronchodilator FEV1/FVC ≥ 0.7 and a smoking history of ≥ 10 pack-years. COPD patients were defined as those with a post-bronchodilator FEV_1_/FVC < 0.7 and a history of smoking ≥ 10 pack-years.

### 2.2 The mouse model of COPD

Twenty female specific pathogen-free (SPF) C57BL/6 mice (6–8 weeks old) weighing 21–24 g were purchased from the Beijing Vital River Experimental Animal Company (Beijing, China). The mice were housed in an SPF facility with free access to sterilised food and water. These mice were exposed to filtered air or cigarette smoke (Baisha cigarettes with filter, Hunan, China) using a nose-only smoke exposure system (SG-300; SIBATA, Saitama, Japan), as described in our previous studies (20, 21). Each mouse was exposed to cigarettes or filtered air for 50 min twice a day with a 20-min smoke-free interval, 5 days a week for 24 consecutive weeks. After 24 weeks of cigarette smoke exposure, the animals were sacrificed and the degree of alveolar damage was measured to evaluate the mouse model. All procedures and protocols followed the “Laboratory Animal Guideline for Ethical Review of Animal Welfare (GB/T 35892-2018)” and the “Guide for the Care and Use of Laboratory Animals: Eighth Edition (2011)” and were approved by the Animal Care Committee of Peking University Third Hospital (batch number: LA2021545), with a duration of approved permit of 4 years, starting from August 2021.

### 2.3 Lung histology and measurement of emphysema

Mouse lung tissues were fixed with 4% paraformaldehyde for 24 h, dehydrated, embedded in paraffin, and sliced into 4-μm sections, followed by haematoxylin and eosin (H&E) staining. Airway space enlargement was quantified using the mean linear intercept (MLI), and the destruction of alveolar walls was quantified using the destructive index (DI), as described in our previous studies (22).

### 2.4 Immunohistochemistry and immunofluorescence of lung tissues

Lung tissues embedded in paraffin were cut into 5-μm sections, and the sections were de-paraffinised, rehydrated, and then incubated in 0.3% H_2_O_2_-CH_3_OH for 15 min to block endogenous peroxidase activity. The sections were then treated with citrate buffer (pH 6.0) or EDTA buffer (pH 9.0) using a microwave oven for 15 min to retrieve antigens, followed by blocking with goat serum for 30 min at room temperature (RT). Subsequently, the tissues were incubated overnight at 4°C with antibodies against C3 (1:2000, Abcam, Cambridge, UK), human and mouse SOD2 (1:500, Cell Signalling Technology, Danvers, MA, USA), mouse cleaved caspase 3 (1:500, Cell Signalling Technology), and human cleaved caspase 3 (1:1000, Cell Signalling Technology). Tissues were incubated with horseradish peroxidase (HRP)-conjugated goat anti-rabbit IgG (ZSGB-Bio, Beijing, China) at RT for 30 min. Slides were visualised using a DAB detection kit (ZSGB-Bio). Images were photographed using a microscope, and then ImageJ software with Colour Deconvolution was installed to split the colour of the immunohistochemical images, which were then analysed using Image-Pro Plus 6.0 software (Media Cybernetics, MD, USA).

For immunofluorescence, human bronchial epithelial (16HBE) cells were fixed in 4% paraformaldehyde at RT for 15 min and then incubated with phosphate-buffered saline (PBS) with 0.2% Triton X-100 for 10 min. Subsequently, the cells were incubated with 1% bovine serum albumin (A8010, Solarbio, Beijing, China) for 30 min to block non-specific antibody binding. Cells were incubated overnight at 4°C with C3 antibodies (1:200, ab97462, Abcam). After washing with PBS, samples were incubated with Alexa 488-labelled goat anti-rabbit secondary antibody (1:800 Jackson Immunoresearch) for 1 h at RT, and then 4, 6-diamidino-2-phenylind-ole dihydrochloride (DAPI) (1:1000, C1002, Beyotime) was added for cellular nuclear staining. C3 expression was evaluated by confocal microscopy (TCS-SP8; Leica Microsystems).

### 2.5 Cell culture

The 16HBE cells were purchased from the Bai Ye Biotechnology Center (SX-C0002, Shanghai, China). Cells were maintained in Dulbecco’s modified Eagle’s medium (SH30022.01, Hyclone, Logan, UT, USA) supplemented with 10% v/v heat-inactivated foetal bovine serum (S12450, Atlanta Biologicals, Flowery Branch, GA) and 1% penicillin/streptomycin (15140122, Gibco, Grand Island, NY, USA). All cell cultures were maintained at 37°C in 5% CO_2_.

### 2.6 Cell treatments

In the cell viability experiments with 16HBE cells, 3% and 5% CSE were used for cell treatment. 16HBE cells and C3-deficient cells (at 70% confluence) were stimulated with medium only or with CSE for 24 h. For the intervention experiments, 16HBE cells were pre-treated with complement C3 protein (A401, QUIDEL, USA) for 2 h, followed by CSE treatment in the presence of C3 for 24 h. The JNK inhibitor SP600125 (catalogue number HY-12041, MCE, Monmouth Junction, NJ, USA) was diluted in dimethyl sulfoxide and stored at a concentration of 10 mM. At the working concentration used, the JNK pathway was inhibited by > 60%. 16HBE cells and C3-deficient cells were pre-treated with 10 μM SP600125 for 1 h and then stimulated with medium only or with CSE for another 24 h.

### 2.7 Short interfering (si)RNA knockdown of C3

For transient transfection, the cells were cultured in six-well culture plates prior to transfection. Either siRNA or a control oligonucleotide (A10001, Gene Pharma, Jiang Su, China) was incubated with 9 μl of Lipofectamine RNAiMAX lipid reagent (13778150, Invitrogen, USA) in 150 μl of Opti-MEM medium (31985070, Gibco) for 5 min at RT. A final volume of 250 μl of C3 siRNA or the control oligonucleotide complex was added to the cell culture plates. After 24 h, CSE was added, and the cells were stimulated for another 24 h. Finally, the cells were harvested for subsequent experiments. The primer sequences were: siControl *Negative control FAM*: 5′-UUCUUCGAACGUGUCACGUTT (forward), 5′-ACGUGACACGUUCGGAGAATT -3′ (reverse); siC3 *C3-homo-66*: 5′-GUCCCAUGUACUCUAUCAUTT (forward); 5′-AUGAUAGAGUACAUGGGACTT-3′ (reverse).

### 2.8 CSE preparation

CSE was prepared as previously described (23). Briefly, five cigarettes (Baisha, China Tobacco Industry Co., Ltd., Hunan, China) were bubbled through 10 ml of medium at a constant velocity (Hyclone, Logan, UT, USA). The solution was filtered through a 0.22 μm filter, which served as the 100% CSE working solution.

### 2.9 Cell viability assessment

Cell viability was tested using a Cell Counting Kit-8 (CCK8) assay kit (KGA317, KeyGEN Biotechnology Co., Ltd., Jiangsu, China). Briefly, cells were treated with medium or different concentrations of CSE for 24 h, washed three times with PBS, and incubated with CCK8 reagent for another 2 h. Finally, the absorbance was assessed using a spectrophotometer at 450 nm.

### 2.10 Western blotting analysis

The 16HBE cells were lysed by RIPA lysis buffer, total proteins were extracted, and the amount was determined with a BCA kit (P0012S, Beyotime Institute of Biotechnology, Beijing, China). Equal quantities of protein (40 µg) were subjected to 7.5%, 10%, or 12.5% SDS-PAGE under a constant voltage of 80 V for 25 min and 120 V for 60 min (WB1102, Biotides, Beijing, China). Subsequently, the protein was transferred to 0.45 μm or 0.22 μm PVDF membranes (Merck-Millipore, Carrigtwohill, Ireland). On a rocking shaker, the membranes were blocked at RT with 5% non-fat milk powder (P1622-1, Applygen, Beijing, China) for 1 h and then incubated with primary antibodies overnight at 4°C, followed by incubation with HRP-conjugated anti-rabbit or anti-mouse antibodies for 1 h at RT. Finally, the membranes were detected using Immobilon Western Chemiluminescent HRP Substrate (Merck Millipore, Carrigtwohill, Ireland). A Tanon 5200 Multi Automatic Chemiluminescence/Fluorescence Image Analysis System was used to visualise the protein expression. Quantitative images were analysed using ImageJ software.

### 2.11 Quantitative real-time reverse transcription PCR (qRT-PCR)

Gene expressions of C3 were determined by qRT-PCR. Total RNA was isolated from the lung tissues of mouse and human COPD patients, and the cultured cells were treated with Trizol reagent (Thermo Fisher Scientific, MA, USA) and the Nano Drop 2000 was used to measure RNA concentration. RNA was then reverse transcribed into cDNA using the HiScript III RT Supermix for qPCR (+gDNA wiper) Kit (R323-01, Vazyme, Nanjing, China). The qPCR reactions were performed on the Applied Biosystems^®^ QuantStudio^®^ 5 in a 20 μl reaction system using the ChamQ Universal SYBR qPCR Master Mix Kit (Q711-02, Vazyme, Nanjing, China).

### 2.12 Cell apoptosis assay

Cell death was determined by flow cytometry in confluent cells that were detached using 0.25% trypsin without EDTA for 3 min. Cells were suspended in cell culture supernatant, centrifuged, resuspended in annexin-binding buffer, washed, and stained with Annexin V Alexa Fluor 488 and Propidium Iodide for 15 min (V13241, ThermoFisher, USA). Flow cytometry was performed using a Beckman CytoFLEX S (Beckman Coulter, Brea, CA, USA). Compensation and data were analysed using Kaluza Analysis version 2.1.

### 2.13 MDA-, SOD-, and ROS measurements

MDA and SOD assay kits (A003-1-2 and A001-3-2, Nanjing Jiancheng Bioengineering Institute, Jiangsu, China) were used according to the manufacturer’s instructions to measure the MDA and SOD content, while the fluorescence dye 2’,7’-dichlorofluorescin diacetate (DCFH-DA; Beyotime Institute of Biotechnology, China) was used to determine the generation of intracellular ROS. Briefly, untreated and C3-deficient cells were plated into 12-well plates and then exposed to 3% and 5% CSE for 24 h. The DCFH-DA solution (20 μM) was added to the cells for 30 min, and the mean fluorescence intensity was measured by flow cytometry.

### 2.14 Statistical analysis

Group data are shown as means ± standard deviation (SD) or median values. Statistical analyses were performed using GraphPad Prism 9. After normality and normality tests, comparisons between the two groups were performed using a two-tailed Student’s t-test or Kolmogorov–Smirnov test. Comparisons between multiple groups were performed using one-way ANOVA with Bonferroni’s multiple comparison test or the Kruskal-Wallis test. *P*-values less than 0.05 were considered statistically significant.

## 3 Results

### 3.1 C3 was highly expressed in AECs of COPD mice

**Figure 1:**
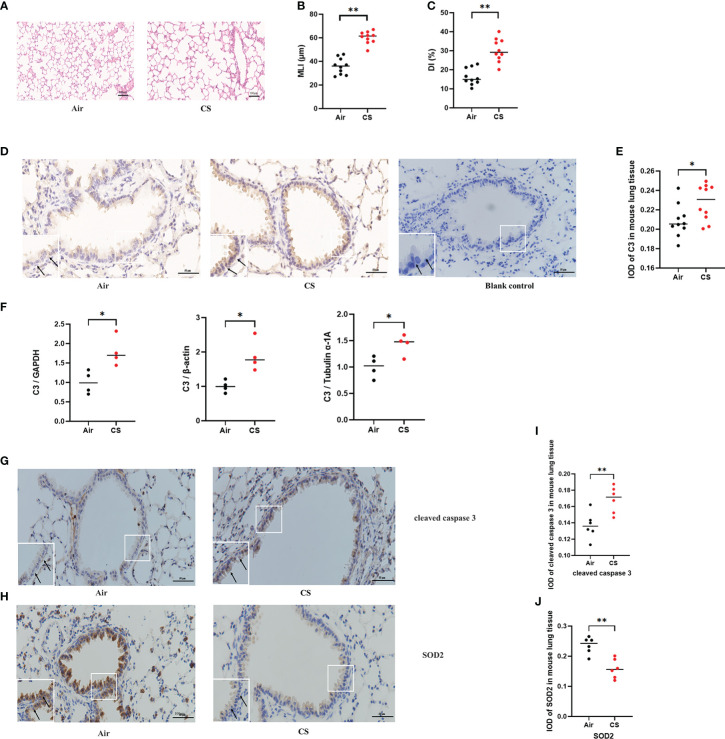
C3, cleaved caspase 3, and SOD2 expression was upregulated in AECs from mouse COPD. **(A)** H&E-stained lung sections. **(B, C)** Mean linear intercept (MLI) and destructive index (DI) was measured. **(D)** Representative immunohistochemical staining of C3 in AECs of the cigarette smoke-exposed group and air-exposed group. **(E)** The integrated optical density (IOD) in immunohistochemistry of C3. N = 10 per group. *P < 0.05. **(F)** C3 mRNA expression in total lung tissue of mice from air-exposed mice and cigarette smoke-exposed mice; N = 4. *P < 0.05. **(G, H)** Representative immunohistochemical staining of cleaved caspase 3 and SOD2 in AECs of air-exposed mice and cigarette smoke-exposed mice. **(I, J)** The IOD in immunohistochemistry of cleaved caspase 3 and SOD2. Data were expressed as group median values. N = 6 per group. **P < 0.01. Original magnification, ×400; scale bars: 50 mM for each figure.

Mice exposed to cigarette smoke for 24 weeks showed lung tissue damage, as demonstrated by increased MLI and DI, which was consistent with changes typical of COPD (**Figures 1A–C**). Immunohistochemistry revealed higher C3 expression in AECs of cigarette smoke-exposed mice (**Figures 1D, E**). In addition, the level of C3 mRNA in the cigarette smoke-exposed group was significantly higher than that in the air-exposed group (**Figure 1F**).

### 3.2 Expression of apoptosis and oxidative stress-related proteins in AECs of COPD mice

A previous study found that exposure to different doses of CSE induced Beas-2B and NHBE cell apoptosis, caspase 3 activation, and oxidative stress, as shown by increased levels of MDA and ROS and decreased levels of SOD (24). To further evaluate the protein expression of oxidative stress and apoptosis markers *in vivo*, we performed immunohistochemistry of lung sections from the mouse model of cigarette smoke-induced COPD. Immunohistochemistry analysis detected increased cleaved caspase 3 and decreased SOD2 protein expression in AECs from cigarette smoke-exposed mice compared to air-exposed mice (**Figures 1G–J**).

### 3.3 C3 was highly expressed in the AECs of COPD patients

**Figure 2:**
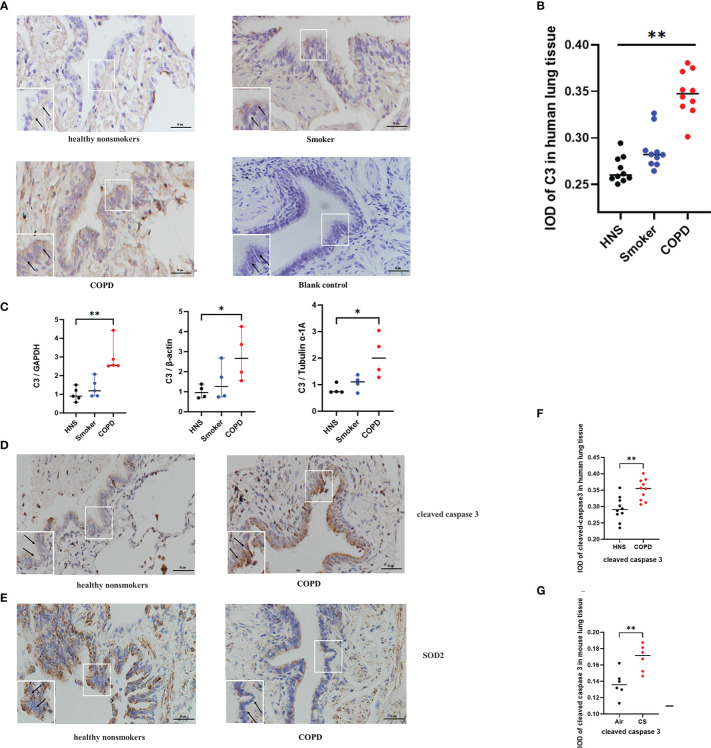
C3, cleaved caspase 3, and SOD2 expression was upregulated in AECs from patients with COPD. **(A)** Representative immunohistochemical staining of C3 in AECs of HNS, smoker, and COPD groups. **(B)** The integrated optical density (IOD) in immunohistochemistry of C3. *N* = 10 per group. **P < 0.01. **(C)** C3 mRNA expression in lung tissue homogenates from HNS, smoker, and COPD groups were detected by qRT-PCR; N ≥ 4. *P < 0.05, **P < 0.01. **(D, E)** Representative immunohistochemical staining of cleaved caspase 3 and SOD2 in lung sections of HNS and patients with COPD. **(F, G)** The IOD in immunohistochemistry of cleaved caspase 3 and SOD2. Data were expressed as group median values. *N* = 10 per group. *P < 0.05, **P < 0.01. Original magnification, ×400; scale bars: 50 mM for each figure.

Previous studies have shown that C3 is expressed at a higher level in AECs from COPD patients than in those from non-COPD subjects (17). To confirm these findings and to further evaluate the expression of C3 in smokers, we assessed the level of C3 expression in AECs of lung tissues from COPD patients, smokers, and HNS controls (n=10 each). The characteristics of the participants are summarised in **Table 1**. Consistent with previous findings, intracellular C3 was detected in the AECs of all the groups. However, C3 expression in AECs was significantly higher in the COPD group than that in the HNS group. Moreover, the expression of C3 in the smoker group was higher than that in the HNS group. C3 was detected in the cytoplasm but not in the nucleus (**Figures 2A, B**). Consistently, C3 gene expression in the COPD group was significantly higher than that in the HNS group (**Figure 2C**). This indicates that intracellular C3 may be derived from endogenous biosynthesis and undergo upregulation in response to cigarette smoke exposure.

**Table 1:** Clinical Characteristics of the study population.

	HNS	Smoker	COPD
Subjects (male, n)	10	10	10
Age (Years	57.30±9.67	61.10±8.16	64.2±5.75
BMI (kg/m2)	24.99±2.24	24.76±1.51	23.33±1.92
FEV1/FVC (%)	79.02±3.43	76.05±4.22	63.76±5.69^*^
FEV1% pred	91.60±4.06	86.22±7.88	67.66±11.52^*^
Smoking index(pack-years)	0	37.50±19.73^#^	51.70±22.51^*^

FEV1 and FEV1/FVC were post-bronchodilator values

Smoking index = average number of cigarettes per day (packs) × number of years of smoking history (years); BMI = weight (kg)/square of height (m^2^). BMI, body mass index; COPD, chronic obstructive pulmonary disease; HNS, healthy non-smoker; FEV1, forced expiratory volume in 1 s; FVC, forced vital capacity

^#^There was a significant difference between smokers and HNS patients

^*^There was significant difference between smokers and COPD patients.

### 3.4 Expression of oxidative stress- and apoptosis-related proteins in the AECs of COPD patients

Consistent with the findings in the mouse model of COPD, cleaved caspase 3 expression was upregulated, and SOD2 expression was downregulated in AECs of COPD patients compared to HNS (**Figures 2D–G**), indicating that oxidative stress and apoptosis were induced in the AECs of COPD patients.

### 3.5 C3 expression was enhanced by CSE exposure in human AECs

**Figure 3:**
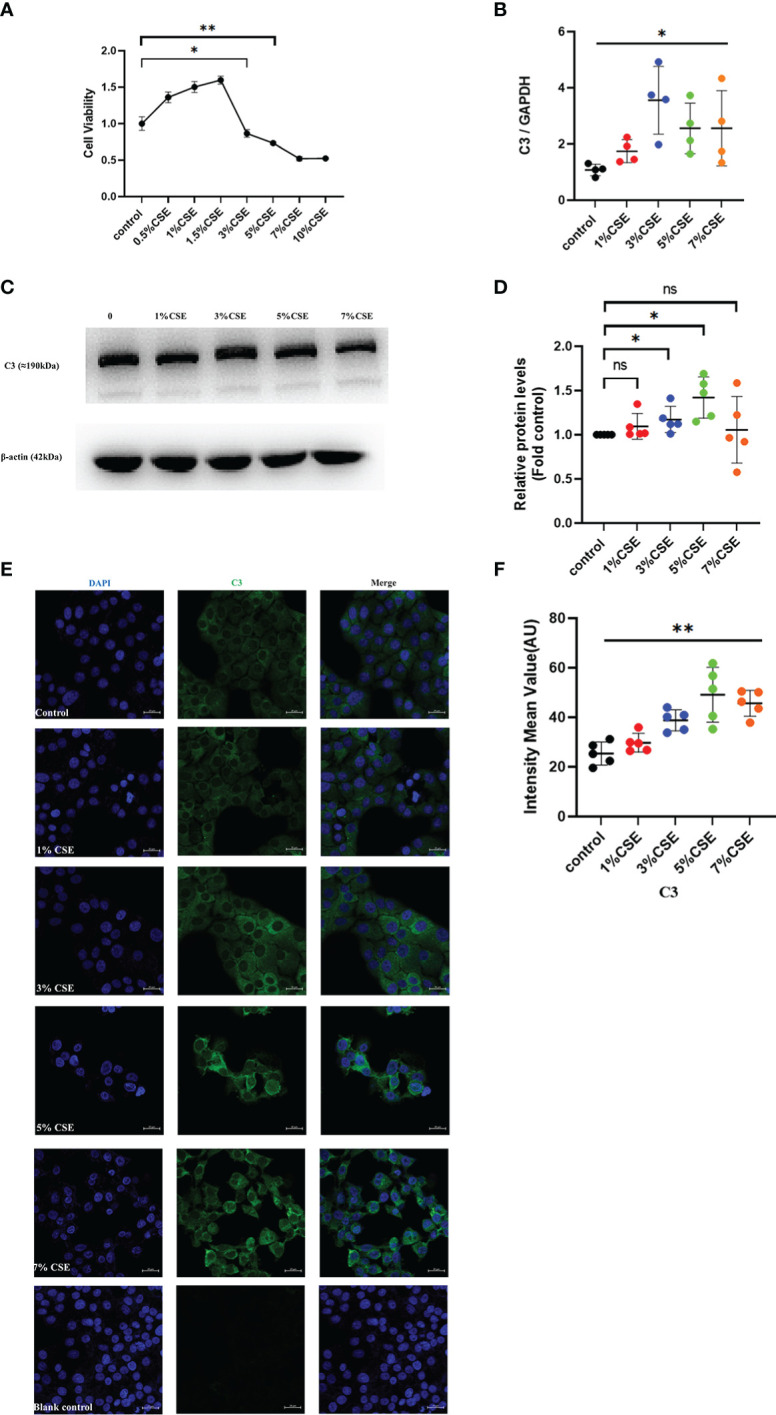
CSE promoted the expression of C3 in lung AECs. **(A)** 16HBE cells were treated with CSE for 24 h, and cellular viability was assessed by CCK8. *P < 0.05. **(B, C)** After 16HBE cells were treated with CSE (1%, 3%, 5%, or 7%) for 24 h, the level of C3 in AECs was measured by qRT-PCR and western blot. *P < 0.05. N = 4 independent experiments. **(D)** Expression of C3 was quantified in 16HBE cells after incubation with different concentrations of CSE for 24 h. *P < 0.05. N = 5 independent experiments. **(E, F)** Expression of C3 in 16HBE cells after CSE incubation for 24 h by immunocytochemistry. Bar: 20 μm. **P < 0.01, ns, no significance.

To confirm the *in vivo* findings of high C3 expression in the AECs of COPD patients and cigarette smoke-exposed mice, we stimulated 16HBE cells with CSE in an *in vitro* culture system. CSE concentrations ≥ 3% showed inhibitory effects on cell activity, while concentrations < 3% appeared to enhance cell viability as determined by CCK8 (**Figure 3A**). Therefore, 16HBE cells were stimulated with various concentrations of CSE (0, 1%, 3%, 5%, or 7%) for 24 h. The qRT-PCR data demonstrated that CSE at different concentrations increased C3 mRNA levels in 16HBE cells (**Figure 3B**). Western blotting results showed that 3% and 5% CSE induced C3 expression in a dose-dependent manner. However, we also found that 1% and 7% CSE induced upregulation of C3 expression, but this was not statistically significant compared to the control group (**Figures 3C, D**). We performed confocal microscopy in cells cultured and passaged in 10% C3-depleted serum, and immunofluorescence (**Figure 3E**) showed that C3 was detected in the 3%, 5%, and 7% CSE groups, while it was hardly detectable in control and 1% CSE groups (**Figure 3F**). These results confirmed that AECs constitutively expressed C3, and CSE promoted C3 expression in these cells.

### 3.6 C3 inhibition enhanced CSE-induced human AEC oxidative stress and apoptosis

**Figure 4:**
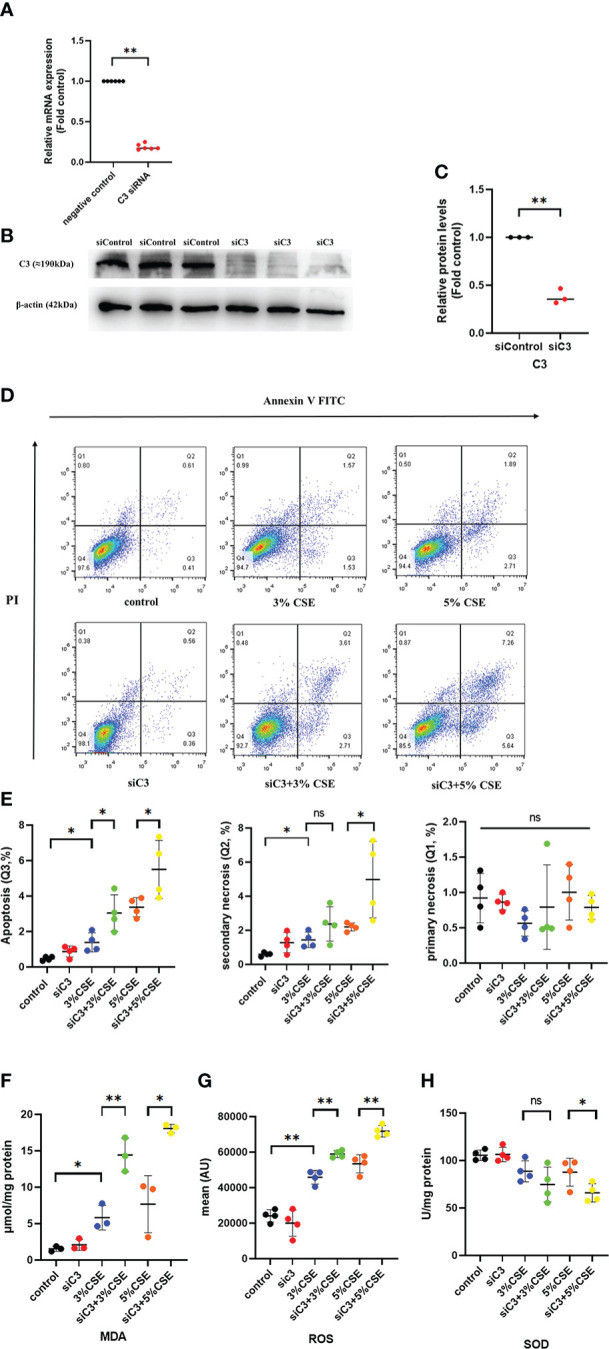
C3 inhibition enhances CSE-induced oxidative stress and apoptosis in AECs. 16HBE cells were transfected with siControl or siRNA targeting human C3 (siC3). **(A)** C3 siRNA reduced C3 mRNA level by 80%. **(B)** Representative immunoblots showing knockdown of C3 proteins using C3-specific siRNA. **(C)** C3 siRNA reduced the C3 protein levels by approximately 70%. **(D)** Cells were left untreated (control) or treated with CSE (3% or 5%) for 24 h. The proportion of apoptotic cells (Q3), secondary necrotic cells (Q2) and primary necrotic cells(Q1) were determined by annexin (x-axis) and propidium iodide (PI, y-axis) staining using flow cytometry. **(E)** Representative analysis plot of cell apoptosis, secondary necrosis and primary necrosis. *P < 0.05. **(F-H)** MDA assay kits were used to measure MDA content, SOD assay kits were used to measure SOD content, and the DCFH-DA method was used to measure ROS production. Data are expressed as mean ± S.D. N ≥ 3 independent experiments. *P < 0.05, **P < 0.01, ns, no significance.

To investigate whether C3 plays a role in AEC death, we used siRNA to inhibit C3 expression in 16HBE cells. C3 silencing was confirmed at both the mRNA and protein levels (**Figures 4A–C**). The results showed that under the two CSE concentrations, the proportion of apoptotic cells(Q3) increased, and this effect was also seen in secondary necrotic cells, i.e., late apoptotic cells (Q2). (**Figures 4D, E**). 16HBE cells with C3KD also showed significantly increased MDA content and ROS production but decreased SOD levels (**Figures 4F–H**). These results suggest that C3 plays an important role in preserving AEC survival and maintaining the oxidative balance.

### 3.7 Exogenous C3 prevented CSE-induced oxidative stress and apoptosis in C3 knockdown human AECs

**Figure 5:**
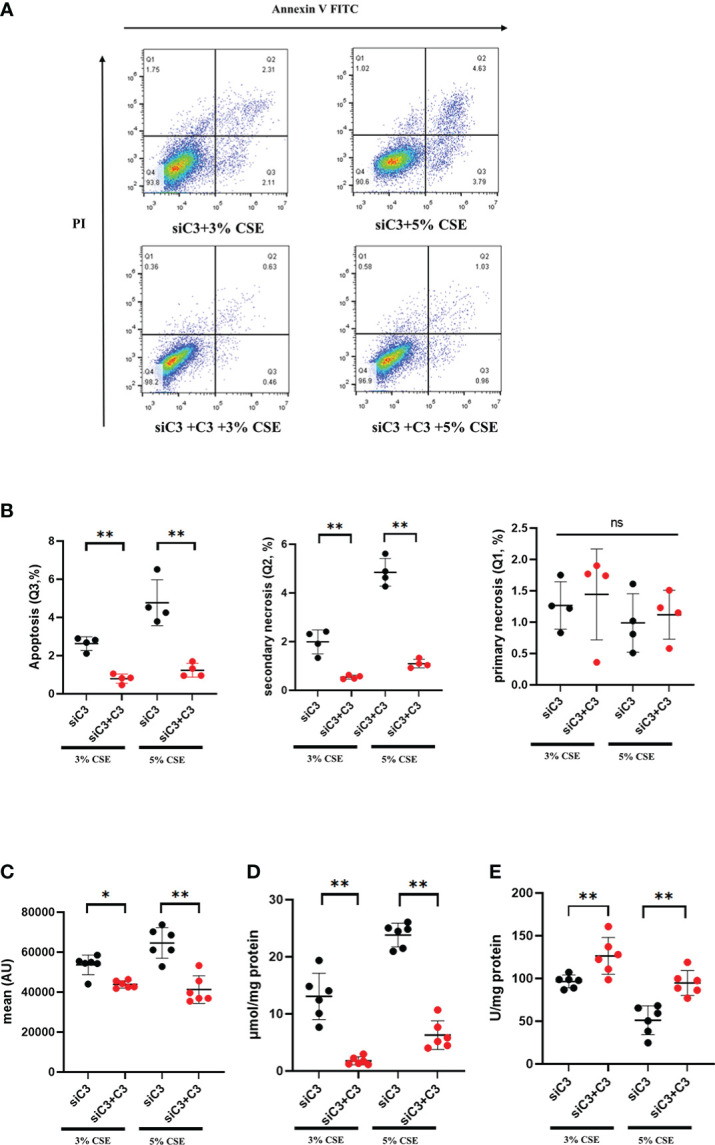
Exogenous C3 prevents CSE-induced oxidative stress and apoptosis in C3 knockdown human AECs. 16HBE cells were transfected with siControl or siRNA targeting human C3. Cells were then treated with CSE (3% and 5% concentration, respectively) in the absence or presence of exogenously added C3 (15 ug/ml) for 24 h. **(A)** The proportion of apoptosis cells(Q3), secondary necrotic cells (Q2) and primary necrotic cells(Q1) were determined using annexin (x-axis) and propidium iodide (PI, y-axis) staining by flow cytometry. **(B)** Representative analysis plot of cell apoptosis, secondary necrosis and primary necrosis. **P < 0.01. **(C-E)** MDA assay kits were used to measure MDA content, SOD assay kits were used to measure SOD content, and the DCFH-DA method was used to measure ROS production. Data were expressed as mean ± S.D. N = 6 independent experiments. *P values < 0.05, **P < 0.01, ns, no significance.

We evaluated whether exogenous C3 could rescue C3-deficient cells from apoptosis and oxidative stress induced by CSE exposure. C3-deficient cells were exposed to CSE in the absence or presence of C3, and oxidative stress and apoptosis were measured. We found that in the presence of C3, CSE-induced apoptosis and secondary necrosis was attenuated in C3-deficient cells (**Figures 5A, B**). Exogenously added C3 reduced oxidative stress, as demonstrated by decreased MDA content and ROS production, but increased SOD levels in C3-deficient cells (**Figures 5C–E**).

### 3.8 The JNK pathway was activated in C3-silenced cells

**Figure 6:**
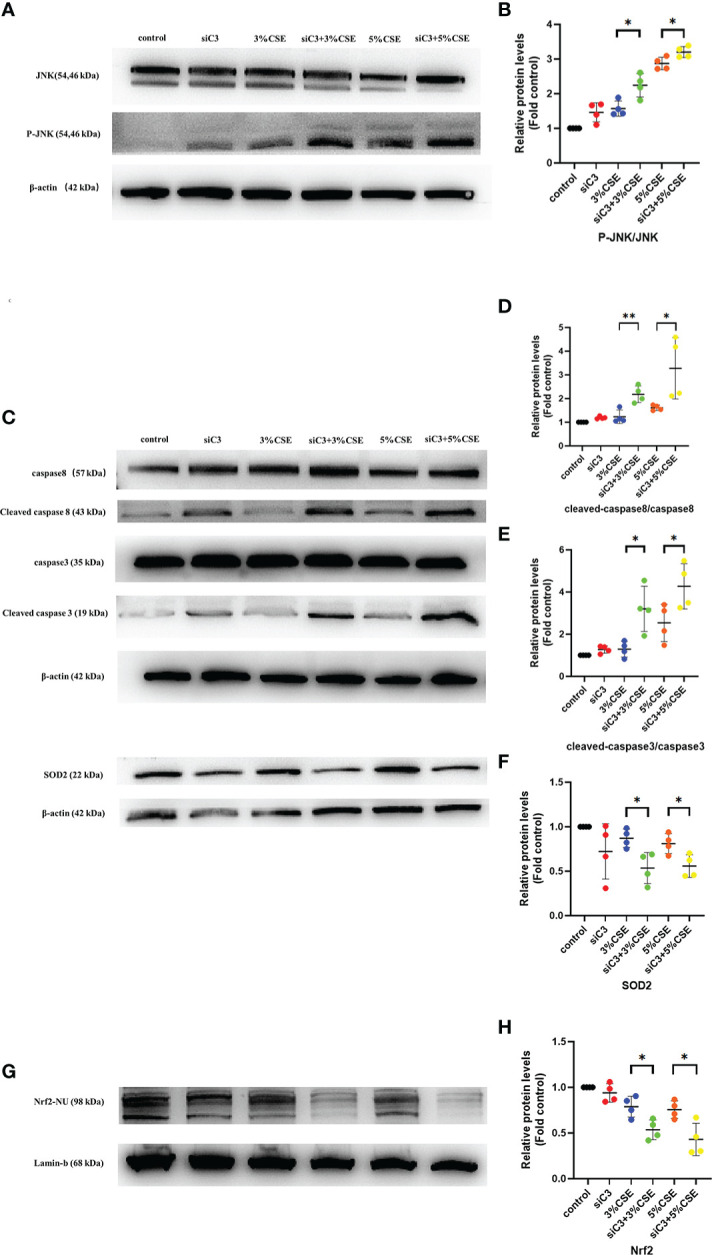
Effects of C3 knockdown on JNK pathway, oxidative stress-, and apoptosis-associated proteins induced by CSE *in vitro*. 16HBE cells were transfected with siControl or siRNA targeting human C3 (siC3). Cells were then left untreated (control) or treated with CSE (3% and 5%, respectively) for 24 h. P-JNK, JNK, cleaved caspase 8, caspase 8, cleaved caspase 3, caspase 3, and Nrf2 inside the nucleus and SOD2 were measured by western blot. **(A, B)** JNK pathway was activated in C3-silenced cells.**(C-H)** C3 inhibition increased the level of cleaved caspase 8 and cleaved caspase 3, and decreased the level of Nrf2 and SOD2 induced by CSE. *P < 0.05. **P < 0.01 N = 4 independent experiments.

Next, we examined the potential mechanisms by which C3 inhibition led to increased oxidative stress and apoptosis in human AECs. The JNK pathway is activated during CSE-induced epithelial cell apoptosis (25, 26), and activation of JNK1 phosphorylation plays an important role in the process of cell death induced by C3 inhibition and/or cytokine exposure (18). Therefore, we investigated whether JNK signalling was disturbed in C3-silenced cells. Furthermore, augmented JNK activation in the context of C3KD and CSE treatment was confirmed by western blotting (**Figures 6A, B**). Other studies have shown that C3 attenuates imiquimod-induced psoriatic skin inflammation by regulating apoptosis-associated proteins (Bak, cleaved caspase 3, and cytochrome c) in mice (27), and colligation of the B cell Ag receptor with the C3-binding CD21/CD19/CD81 costimulatory complex enhances the escape of human B cells from Fas-induced death by regulating the expression of molecules required for death-inducing signal complex formation (Fas, FADD, and caspase 8) (28). Therefore, we further measured apoptosis-associated proteins in CSE-induced 16HBE cells by western blotting. Our results showed that CSE stimulation markedly increased the levels of cleaved caspase 8 and cleaved caspase 3. More notably, the levels of cleaved caspase 8 and cleaved caspase 3 were higher in C3KD cells than in CSE-treated cells (**Figures 6C–E**). Thus, C3KD increased the concentrations of CSE-induced apoptosis proteins, as evaluated by the cleavage of caspase 8 and caspase 3 (**Figures 6C–E**).

Activation of the Nrf2 pathway leads to the expression of antioxidative molecules such as SOD1 and SOD2, which play an anti-inflammatory role in COPD (29). Therefore, we measured the Nrf2/SOD2 axis by western blotting. We found that both concentrations of CSE exposure reduced the expression of Nrf2 in the nucleus and downstream SOD2 in the cytoplasm. Moreover, C3KD increased CSE-induced oxidative stress in AECs (**Figures 6C, F–H**).

### 3.9 JNK inhibition protected against C3 deficiency-induced oxidative stress and apoptosis

**Figure 7:**
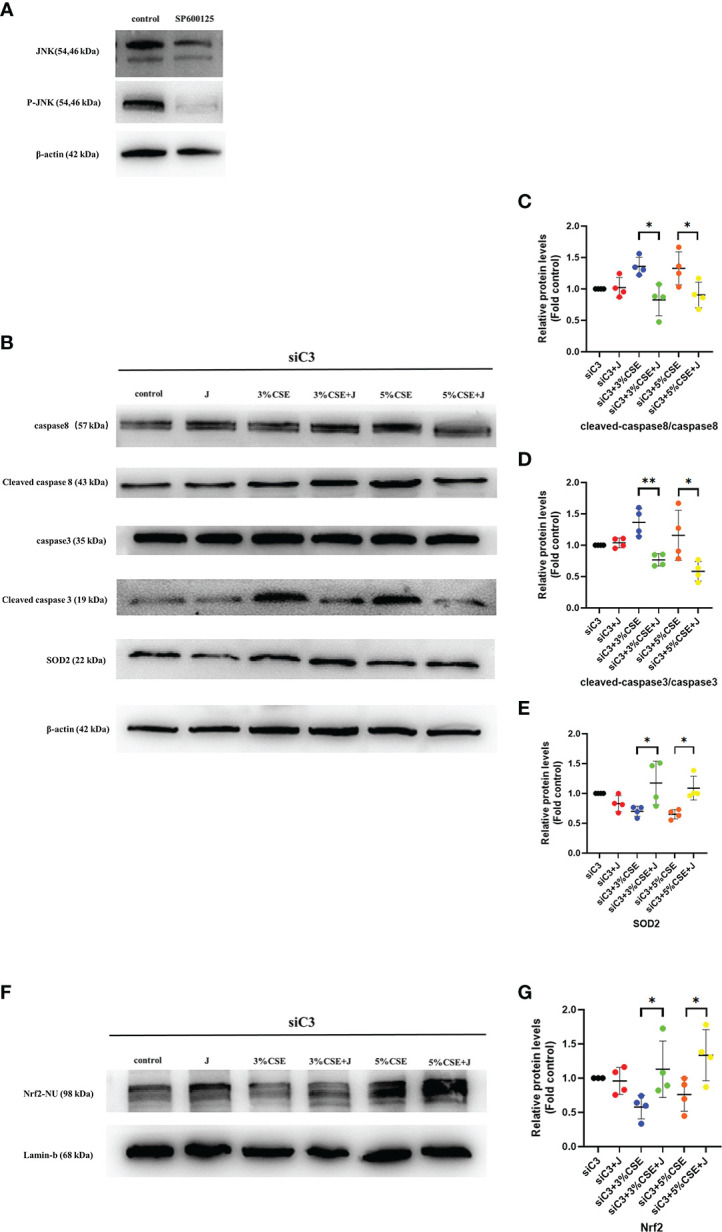
Pharmacological inhibition of the JNK pathway with SP600125 rescued CSE-induced oxidative stress and apoptosis in C3KD cells. C3KD cells were treated with SP600125 (10 μM) for 1 h, then stimulated with 3% or 5% CSE for another 24 h. **(A)** SP600125 significantly inhibited the level of JNK pathway activation by western blot. **(B-G)** Pharmacological inhibition with SP600125 rescued increased protein levels of cleaved caspase 8 and cleaved caspase 3 induced by CSE, while mitigating decreased protein levels of Nrf2 inside the nucleus and SOD2 induced by CSE. *P < 0.05, **P < 0.01. N = 4 independent experiments.

To determine whether JNK activation contributed to the AEC death observed in C3KD cells, we silenced C3 and JNK in parallel in 16HBE cells. JNK inhibition was achieved using the chemical JNK inhibitor SP600125 (**Figure 7A**). JNK inhibition prevented CSE-induced oxidative stress and apoptosis in C3KD cells, as evaluated by oxidative stress and apoptosis-related pathway proteins (**Figures 7B–G**). These data suggest that JNK phosphorylation is a pivotal component of the cell death process induced by C3 inhibition and CSE exposure.

## 4 Discussion

Many immune and non-immune cells produce complement proteins (16, 30). Recent studies have demonstrated that human AECs have intracellular C3 stores primarily obtained by biosynthesis, and intracellular C3 is present at relatively high levels in lung AECs of end-stage COPD patients (17). However, the role of C3 and the cellular and molecular pathways involved in COPD remain largely unknown. In the present study, we first confirmed the previous findings of increased intracellular C3 in the AECs of COPD patients and further revealed upregulation of C3 in a well-established mouse model of COPD induced by long-term cigarette smoke exposure. The results of the *in vitro* experiments showed that human AECs constitutively expressed C3 derived from biosynthesis, and CSE exposure promoted C3 expression. Oxidative stress and apoptosis play important roles in the pathogenesis of COPD caused by cigarette smoke exposure (31), and there is evidence showing increased oxidative stress and AEC apoptosis in the lungs of smokers and COPD patients, resulting in airspace enlargement and alveolar wall damage (32, 33). To the best of our knowledge, this is the first study to demonstrate that C3 deficiency exacerbated CSE-induced oxidative stress and apoptosis in 16HBE cells, and exogenous C3 partially rescued CSE-induced cell death and oxidative stress in C3KD cells. We further demonstrated that C3 exerted pro-survival and antioxidant effects through JNK inhibition. These data revealed a novel mechanism for the pathogenesis of COPD associated with cigarette smoking.

The complement system acts as a bridge between innate and adaptive immunity and plays an important role in defence against invading pathogens (34). The activation and function of complement proteins are not limited to the extracellular space but also occur within the cell, such as metabolic reprogramming, proliferation, homeostasis, and survival (35, 36). The cytoprotective properties of C3 have been observed in other organ systems. Studies have found that in the liver, complement C3a and C5a are critical for cell survival during hepatocyte regeneration (37). Moreover, internalised C3 protected cells from stress-induced death, and intracellular production of C3 promoted cell survival, while apoptosis was increased after C3 knockdown with siRNA in islet β-cells (17, 18). In this study, we stimulated 16HBE cells *in vitro* with two concentrations of CSE and found that 3% and 5% CSE induced an increase in oxidative stress and apoptosis. More importantly, we observed that C3 deficiency resulted in increased CSE-induced oxidative stress and apoptosis in human AECs, and exogenously added C3 rescued C3-deficient cells from oxidative stress and apoptosis. This led to the conclusion that C3 may promote AEC survival *via* intracellular production as well as *via* a paracrine pathway.

The JNK pathway is activated and plays a role in CSE-induced epithelial apoptosis (38). Upon activation, JNK directly inhibits the anti-apoptotic protein, Bcl2, thereby promoting apoptosis. It can also translocate to the mitochondrial membrane and release cytochrome C, which, as an important pro-apoptosis factor, promotes the activation of the caspase cascade reaction, leading to protein cleavage and, ultimately, apoptosis (39, 40). JNK acts as a crucial pro-cell damage pathway in endothelial and epithelial cells exposed to CSE (38, 41). In the current study, we found that JNK was activated in C3-silenced cells, leading to higher levels of cleaved caspase 8 and cleaved caspase 3 and subsequent activation of apoptosis. Moreover, abundant evidence has confirmed that the Nrf2/SOD2 signalling pathway is involved in oxidative stress responses (42, 43). Recent studies have also found that silychristin A activates the Nrf2-HO1/SOD2 pathway to reduce apoptosis in GLUTag cells (44). However, the role of C3 in CSE-induced oxidative stress in epithelial cells has not yet been demonstrated. A previous study showed that C3 (H_2_O) internalised by BEAS-2B cells rescued the cells from H_2_O_2_-induced stress-associated cell death (17); however, the biological relevance of this *in vitro* model of oxidative stress requires further investigation. The present study confirmed that CSE treatment inhibited the expression of Nrf2 and downstream SOD2 proteins. Interestingly, we also observed concomitant activation of JNK in C3-silenced cells, resulting in higher levels of Nrf2 and SOD2 expression. JNK inhibition completely prevented CSE-induced upregulation of oxidative stress- and apoptosis-related proteins, indicating that activation of the JNK pathway contributes to the deleterious effects of C3 inhibition.

Although our data suggest that both extracellular and intracellular C3 activation are important for AEC survival, the mechanism by which C3 is activated in the lung environment of COPD remains to be clarified. It has been recently proposed that IL-17A induces protein and mRNA regulation of C3, and limiting complement activation by neutralising IL-17A is a potential mechanism for ameliorating pulmonary fibrosis (45). Epithelium-derived IL-17A plays an important role in cigarette smoke-induced inflammation and mucus overproduction (46). Therefore, it is conceivable that components of the complement system secreted by the epithelium culminate in C3 activation, which protects AECs against pro-inflammatory assaults. This protection may be sufficient to prevent AEC death during mild activation of the innate immune response but may not be sufficient to prevent AEC death in the context of a prolonged autoimmune attack. However, the exact role and potential mechanisms of C3 in COPD need to be explored in future studies.

In conclusion, we provide evidence that C3 is an important factor in human AECs modulated by cigarette smoke exposure and that it exerts anti-oxidative stress and pro-survival effects in mouse and human AECs. Furthermore, C3 deficiency facilitated JNK activation and decreased the death receptor and Nrf2/SOD2 pathway activation, while JNK inhibition protected against C3 deficiency-induced oxidative stress and apoptosis. Thus, this study demonstrated a protective role of C3 against cigarette smoke-induced AEC oxidative stress and apoptosis through mediation of the JNK pathway, revealing a potentially novel mechanism by which the complement system participates in the pathogenesis of COPD associated with cigarette smoking.

## Data availability statement

The original contributions presented in the study are included in the article/**Supplementary Material**. Further inquiries can be directed to the corresponding authors.

## Ethics statement

The studies involving human participants were reviewed and approved by the ethics review committee of Peking University Third Hospital. The patients/participants provided their written informed consent to participate in this study. The animal study was reviewed and approved by Animal Care Committee of Peking University Third Hospital.

## Author contributions

Conception and design: YP, JZ, and YS. Data acquisition: JQ, YP, DL, and YS. Help with animal experiments: YP, YR, and DL. Analysis and interpretation: YP, JZ, XG, YC, YL, and YS. Drafting the manuscript for important intellectual content: YP, DL, and YS. All authors have contributed to the manuscript and approved the submitted version.
