# Supplementary material for: Complement component 3 protects human bronchial epithelial cells from cigarette smoke-induced oxidative stress and prevents incessant apoptosis
Source: Front Immunol. 2022 Dec 20;13:1035930. doi: 10.3389/fimmu.2022.1035930 (PMC9807617; doi:10.3389/fimmu.2022.1035930)
Supplement: Supplementary file 2 [file Table_1.doc]

**Table E1** Primers sequences used in RT-PCR

**Forward**  **Reverse**

human *C3*: TCACCGTCAACCACAAAGCTGCTACC TTTCATAGTAGGCTCGGATCTTCCA

mouse *C3*: ACCTTACCTCGGCAAGTTTCT TTGTAGAGCTGCTGGTCAGG

human *GAPDH*: AAGAAGGTGGTGAAGCAGG GAAGGTGGAAGAGTGGGAGT

mouse *GAPDH*: AAATGGTGAAGGTCGGTGTGAAC CAACAATCTCCACTTTGCCACTG

human *β-actin*: CATGTACGTTGCTATCCAGGC CTCCTTAATGTCACGCACGAT

mouse *β-actin*: GTACCACCATGTACCCAGGC AACGCAGCTCAGTAACAGTCC

human *Tuba1a*: GAAGTTCGCACTGGCACCTACC TCGGGCATAGTTATTGGCAGCATC

mousse *Tuba1a*: CGGCTCTCTGTGGATTACGGAAAG TGGTGTGGGTGGTGAGGATGG
